# Racial differences in six major subtypes of melanoma: descriptive epidemiology

**DOI:** 10.1186/s12885-016-2747-6

**Published:** 2016-08-30

**Authors:** Yu Wang, Yinjun Zhao, Shuangge Ma

**Affiliations:** 1 School of Statistics and The center for Applied Statistics, Renmin University of China, 59 Zhongguancun Ave., Beijing, 100872 China; 2 School of Public Health, Yale University, 60 College ST, LEPH 206, New Haven, CT 06520 USA; 3 VA Cooperative Studies Program Coordinating Center, West Haven, CT USA

**Keywords:** Melanoma, Racial difference, Subtype, SEER

## Abstract

**Background:**

Melanoma accounts for the majority of skin cancer deaths. It has over thirty different subtypes. Different races have been observed to differ in multiple aspects of melanoma.

**Methods:**

SEER (Surveillance, Epidemiology, and End Results) data on six major subtypes, namely melanoma in situ (MIS), superficial spreading melanoma (SSM), nodular melanoma (NM), lentigo maligna melanoma (LMM), acral lentiginous melanoma malignant (ALM), and malignant melanoma NOS (NOS), were analyzed. The racial groups studied included NHW (non-Hispanic white), HW (Hispanic white), Black, and Asian/PI (Pacific Islanders). Univariate and multivariate analysis was conducted to quantify racial differences in patients’ characteristics, incidence, treatment, and survival.

**Results:**

Significant racial differences are observed in patients’ characteristics. For all subtypes except for ALM, NHWs have the highest incidence rates, followed by HWs, while Blacks have the lowest. For ALM, HWs have the highest rate, followed by NHWs. In stratified analysis, interaction between gender and race is observed. For the first five subtypes and localized and regional NOS, the dominating majority of patients had surgery, while for distant NOS, the distribution of treatment is more scattered. Significant racial differences are observed for distant ALM and NOS. For MIS, SSM, NM, LMM, and ALM, there is no significant racial difference in survival. For NOS, significant racial differences in survival are observed for the localized and regional stages, with NHWs having the best and Blacks having the worst five-year survival rates.

**Conclusions:**

Racial differences exist for the six major melanoma subtypes in the U.S. More data collection and analysis are needed to fully describe and interpret the differences across racial groups and across subtypes.

## Background

Melanoma is the most dangerous type of skin cancer. In 2015, it is estimated that there were 73,870 new cases, and an estimated 9,940 people died of this disease [1]. It represents 4.5 % of all new cancer cases. The incidence of melanoma has been steadily rising since 1975 in the U.S. [2]. Melanoma has over thirty different subtypes with significantly different behaviors. In this article, the focus is on the following six most major subtypes. Melanoma in site (MIS) is an early form of melanoma with atypical melanocytes confined to the epidermis. Superficial spreading melanoma (SSM) is more common for the 30–50 years old, often on the trunk, and in women often on the legs. Nodular melanoma (NM) is more common for the 40–60 years old and twice as common in men. It has no horizontal growth phase and rapid vertical growth. Lentigo maligna melanoma (LMM) is more common for the 50–80 years old, especially with sun-damaged skin. It develops on the face in 90 % of cases. Acral lentiginous melanoma (ALM) presents up to 75 % of melanomas in non-Caucasian patients and occurs on acral surfaces. The last major subtype studied is malignant melanoma, NOS.

It has been suggested that there exist racial differences in multiple aspects of melanoma. Overall, Whites have a higher risk and poorer prognosis. Melanoma occurs more commonly in unusual anatomic sites (e.g., palms and soles) in minority populations than in Whites [3]. With rare occurrence and unusual presentation, the diagnosis of melanoma is often delayed in minorities, leading to more advanced stages. A few studies have been conducted, examining racial difference in melanoma. Examples include that by Du and others [4], which linked the NLMS (National Longitudinal Mortality Studies) and SEER (Surveillance, Epidemiology, and End Results) databases and examined the effects of individual-level socioeconomic factors on racial disparities in receiving treatment and survival. Another study examined racial differences in overall and melanoma-specific survival, stratified by receipt of surgical treatment and by specific types of surgical treatment [5]. Cormier and others [6] analyzed SEER data and quantified racial differences in clinicopathologic factors and survival for cutaneous melanoma patients. Results in the literature have not always been consistent. For example, Reintgen and others [7] reported differences in stage-specific melanoma outcomes between Blacks and Whites, however, Hemmings and others [8] reported no differences in outcomes in non-Whites versus Whites who were stratified by stage at initial diagnosis.

Despite the aforementioned efforts, to date, racial differences in melanoma still have not received sufficient attention. The goal of this study is to fill this knowledge gap and systematically describe racial differences for the six most major subtypes of melanoma using SEER data. Studying racial difference can assist better diagnosis, tailored treatment, and elimination of racial disparity. Analyzing and directly comparing multiple subtypes can provide valuable insights beyond single-subtype analysis [9]. This study differs from and complements the existing literature in multiple aspects. First, it analyzes the six major subtypes separately and can better accommodate cancer heterogeneity than studies that analyze melanoma overall [10]. Second, it analyzes patient characteristics, incidence, treatment, as well as survival for four major racial groups, and can be more comprehensive than those that focus on one specific aspect and fewer racial groups [5]. Third, different subtypes are analyzed on the same ground using the same techniques. Some of the existing studies have also conducted subtype analysis [9]. However, as they analyzed different study populations and adopted different statistical techniques, the results so generated may not be fully comparable.

## Methods

### Source population

The population-based sample was obtained from SEER (Surveillance, Epidemiology, and End Results) [11], which is the most comprehensive population-based cancer database in the U.S., containing data from eighteen regional and state registries. SEER has multiple registry groupings for analysis, which cover different numbers of regions and different time period. SEER 9, 13, and 18, which are analyzed in this study, cover approximately 9.5, 14, and 28 % of the U.S. population, respectively [12].

For each case, the first matching record was identified for analysis. Incident cases of melanoma of the skin – defined using ICD-O-3 site codes C440-449 and histology codes 8720-8790 – were selected. The histology codes were grouped for analysis as follows: MIS (ICD-O-3 code 8720/2), NM (ICD-O-3 code 8721/3), LMM (ICD-O-3 code 8742/3), SSM (ICD-O-3 code 8743/3), ALM (ICD-O-3 code 8744/3), and NOS (ICD-O-3 code 8720/3).

Different registry groupings were used for different analysis to maximize sample size. Specifically, for the analysis of patients’ clinicopathologic features, SEER 9 contains data on cancers diagnosed between 1973 and 2011. Information is available on gender, marital status, age at diagnosis, age group, anatomic site, thickness of tumor, presence of satellite nodules, ulceration, lymph node extension, stage, treatment, and type of surgery. More details are available in Table 1. The variable “anatomic sites” is defined using ICD-O-3 [13]. Anatomic body sites include skin of the face, head, and neck (C44.0–44.4), trunk (C44.5, including back, abdomen, and chest), upper extremity (C44.6), lower extremity (C44.7), and all “other or unknown” body sites which are combined into a single category. Four variables, including “satellite tumors” (1973–1982), “4-Digit Extent of Disease (EOD 4)-extension” (1983–1987), “10-Digit Extent of Disease (EOD 10)-extension” (1988–2003) and “clinical stage (CS) lymph nodes” (2004), are recoded to form the three-category satellite nodule variable. Three variables, including “type of melanoma” (1973–1982), “extension” (1988–2003), and “CS site specific factor 2 ulceration” (2004), are recoded to form the three-category skin ulceration variable. Skin ulceration status was not coded between 1983 and 1987, therefore, all 1983–1987 cases are coded as having “unknown” for ulceration. Five variables, including “regional lymph node involvement” (1973–1982), “distant lymph nodes” (1973–1982), “EOD 4 lymph nodes” (1983–1997), “EOD 10 lymph nodes” (1988–2003), “CS lymph nodes” (2004), are recoded to categorize the extent of lymph node involvement. Treatment is analyzed both as a patient’s characteristic and as a cancer response variable. For melanoma, removal by surgery is the most common treatment. Other options include immunotherapy, biologic therapy, radiation, chemotherapy, and others. SEER contains information on surgery and radiation but no other treatments. As a result, patients in the “no surgery or radiation” category might receive other types of treatment, but this information is not available. For the analysis of incidence, SEER 13 contains detailed race and incidence information for cancers diagnosed between 1992 and 2011. For the analysis of survival, SEER 18 contains information for cancers diagnosed between 1973 and 2006 and followed up to 12/31/2011.

**Table 1:** Patients’ characteristics and clinicopathologic features

	Melanoma in situ					Superficial spreading melanoma					Nodular melanoma				
	NHW (*n* = 87852)	HW (*n* = 1890)	Black (*n* = 222)	Asian/PI (*n* = 180)	*P*	NHW (*n* = 84790)	HW (*n* = 1993)	Black (*n* = 218)	Asian/PI (*n* = 364)	*P*	NHW (*n* = 19260)	HW (*n* = 689)	Black (*n* = 106)	Asian/PI (*n* = 160)	*P*
Gender					<0.001					<0.001					<0.001
Male	54.7	36.0	45.0	44.6		53.3	35.6	42.2	49.2		61.6	51.8	44.3	56.3	
Female	45.3	64.0	55.0	55.4		46.7	64.4	57.8	50.8		38.4	48.2	55.7	43.8	
Marital Status					<0.001					<0.001					<0.001
Single	12.6	15.4	22.1	18.8		15.4	18.8	26.3	23.9		14.9	21.3	37.0	17.6	
Married	73.9	70.8	54.7	67.7		71.2	67.7	48.1	62.7		63.5	58.5	34.8	62.0	
Separated/divorced/widowed	13.5	13.8	23.3	13.5		13.4	13.5	25.6	13.4		21.6	20.2	28.3	20.4	
Age at diagnosis	60.5 ± 15.9	55.6 ± 16.9	59.2 ± 16.2	55.0 ± 17.9	<0.001	54.7 ± 16.7	49.2 ± 16.5	56.0 ± 17.7	52.7 ± 17.3	<0.001	62.1 ± 17.7	55.9 ± 19.1	62.7 ± 18.1	60.3 ± 20.1	<0.001
Anatomic Site					<0.001					<0.001					<0.001
Face/head/neck	25.7	28.6	18.0	22.3		13.7	14.4	9.6	11.0		22.9	22.5	15.1	16.9	
Trunk	30.9	24.1	12.6	26.0		39.0	33.9	29.8	36.0		32.1	28.3	18.9	26.3	
Upper Extremity	25.9	23.9	24.8	22.8		25.7	22.9	17.0	18.7		27.1	20.8	12.3	13.8	
Lower Extremity	17.0	23.0	44.1	29.0		21.0	28.4	42.2	33.5		17.4	28.0	51.9	43.1	
Other	0.6	0.5	0.5	0		0.5	0.4	1.4	0.8		0.6	0.4	1.9	0	
Thickness of tumor (mm)	0.80 ± 1.83	0.78 ± 1.85	1.02 ± 1.31	0.68 ± 0.88	0.980	0.92 ± 1.09	1.01 ± 1.20	1.51 ± 2.11	1.36 ± 1.96	<0.001	3.27 ± 2.51	3.93 ± 3.02	4.22 ± 3.18	4.23 ± 3.04	<0.001
Satellite nodules										0.012					0.280
No	-	-	-	-		97.0	97.2	95.9	95.3		93.5	93.9	88.7	95.6	
Yes	-	-	-	-		0.6	1.1	0.9	0.8		3.6	3.5	4.7	3.1	
Unknown	-	-	-	-		2.4	1.7	3.2	3.8		3.0	2.6	6.6	1.3	
Ulceration					0.242					0.001					0.001
No	92.2	91.7	94.1	91.1		86.7	87.6	83.5	87.4		59.8	56.0	50.9	56.3	
Yes	0.5	0.4	0.9	0		4.9	6.0	8.3	6.6		31.4	38.3	37.7	35.6	
Unknown	7.3	7.9	5.0	8.9		8.4	6.4	8.3	6.0		8.8	5.7	11.3	8.1	
Lymph node extension					0.866					<0.001					<0.001
None	99.9	99.8	99.5	100.0		72.7	78.8	67.0	76.9		63.3	60.4	52.8	58.1	
Regional	0	0	0	0		3.2	4.7	5.0	6.6		16.6	25.3	18.9	25.0	
Distant	0	0	0	0		0.1	0.2	0	0		0.9	2.0	5.7	1.9	
Unknown	0.1	0.2	0.5	0		24.0	16.3	28.0	16.5		19.2	12.3	22.6	15.0	
Stage					0.996					<0.001					<0.001
In situ	100.0	100.0	100.0	100.0		-	-	-	-		-	-	-	-	
Localized	-	-	-	-		92.7	90.8	86.7	87.9		62.1	51.1	43.4	52.5	
Regional	-	-	-	-		5.4	7.4	9.6	9.6		32.0	40.1	38.7	41.3	
Distant	-	-	-	-		0.4	0.8	0.9	0.8		4.1	7.1	14.2	5.6	
Unstaged	0.0	-	-	-		1.5	1.1	2.8	1.6		1.9	1.7	3.8	0.6	
Treatment					0.152					0.005					0.033
No treatment	4.1	5.8	5.0	4.2		1.6	2.0	1.4	2.2		1.5	1.6	1.9	1.3	
Surgery	95.1	93.7	94.6	95.3		97.1	97.1	95.4	95.6		94.7	94.8	90.6	93.8	
Radiation	0.1	0.1	0	0.2		0.1	0	0.5	0		0.2	0	0	1.3	
Radiation & Surgery	0.4	0.3	0	0		0.9	0.8	1.4	1.9		3.3	3.2	5.7	3.1	
Unknown	0.2	0.2	0.5	0.2		0.4	0.1	1.4	0.3		0.4	0.4	1.9	0.6	
Type of surgery					<0.001					<0.001					<0.001
No surgery	4.2	5.9	5.1	4.5		1.7	2.1	1.6	2.3		1.8	1.7	2.0	2.1	
Biopsy	65.8	63.9	51.6	55.8		43.0	43.6	37.0	42.9		32.5	35.1	34.3	32.9	
Wide excision	29.8	29.9	40.0	39.3		55.0	53.5	59.3	54.2		64.3	57.3	57.6	60.3	
Amputation	0.1	0.2	2.8	0.5		0.1	0.4	2.1	0.3		0.5	3.5	3.0	4.1	
Surgery NOS	0.1	0.1	0.5	0		0.3	0.5	0	0.3		0.9	2.4	3.0	0.7	
Survival time (month)	300.7 ± 3.0	355.9 ± 13.1	287.8 ± 15.3	325.6 ± 19.6	<0.001	298.7 ± 1.2	305.8 ± 6.3	277.1 ± 17.5	300.2 ± 13.6	0.001	168.5 ± 1.8	160.3 ± 9.6	106.9 ± 14.3	145.8 ± 16.6	0.033
	Lentigo maligna melanoma					Acral lentiginous melanoma, malignant					Malignant melanoma, NOS				
	NHW (*n* = 17226)	HW (*n* = 250)	Black (*n* = 46)	Asian/PI (*n* = 69)	*P*	NHW (*n* = 1822)	HW (*n* = 300)	Black (*n* = 215)	Asian/PI (*n* = 168)	*P*	NHW (*n* = 127924)	HW (*n* = 3807)	Black (*n* = 788)	Asian/PI (*n* = 861)	*P*
Gender					<0.001					0.138					<0.001
Male	67.2	54.4	54.3	56.5		46.5	46.3	40.9	53.0		57.5	42.6	48.4	48.5	
Female	32.8	45.6	45.7	43.5		53.5	53.7	59.1	47.0		42.5	57.4	51.6	51.5	
Marital Status					0.283					<0.001					<0.001
Single	8.3	11.6	6.3	7.4		12.9	15.2	17.3	10.2		14.8	20.5	28.0	16.2	
Married	71.4	71.1	59.4	74.1		65.7	59.8	42.6	67.5		68.8	63.1	42.3	66.8	
Separated/divorced/widowed	20.3	17.4	34.4	18.5		21.5	25.0	40.1	22.3		16.4	16.3	29.7	17.0	
Age at diagnosis	69.7 ± 12.6	68.0 ± 14.5	65.3 ± 13.2	66.3 ± 16.8	0.001	63.6 ± 16.3	61.4 ± 16.8	65.5 ± 16.4	64.1 ± 16.1	0.034	58.9 ± 17.2	53.5 ± 18.2	60.3 ± 18.5	57.0 ± 19.3	<0.001
Anatomic Site					<0.001					0.010					<0.001
Face/head/neck	60.3	70.4	37.0	46.4		1.8	1.3	0.5	0		18.6	17.5	9.9	10.6	
Trunk	15.3	11.6	8.7	15.9		2.2	1.0	0	0		32.2	24.4	15.4	20.0	
Upper Extremity	19.3	11.6	6.5	14.5		20.2	16.3	15.3	19.0		23.7	19.7	11.3	17.5	
Lower Extremity	4.4	6.0	45.7	23.2		75.6	81.0	83.7	79.8		17.5	26.6	43.8	33.6	
Other	0.6	0.4	2.2	0		0.2	0.3	0.5	1.2		8.0	11.7	19.7	18.4	
Thickness of tumor (mm)	0.70 ± 1.00	0.73 ± 0.85	1.98 ± 2.59	0.96 ± 1.31	<0.001	2.12 ± 2.08	2.38 ± 2.27	2.53 ± 2.39	2.99 ± 2.47	<0.001	1.10 ± 1.47	1.53 ± 1.95	2.42 ± 2.63	1.75 ± 2.08	<0.001
Satellite nodules					<0.001					0.016					<0.001
No	96.1	97.2	89.1	89.9		94.5	92.7	93.5	86.9		89.3	85.5	79.1	80.8	
Yes	0.5	0	8.7	2.9		2.8	3.7	3.7	6.5		0.8	1.1	2.7	1.5	
Unknown	3.4	2.8	2.2	7.2		2.7	3.7	2.8	6.5		9.9	13.4	18.3	17.7	
Ulceration					<0.001					0.025					<0.001
No	88.8	85.6	67.4	82.6		70.5	65.3	62.3	60.7		78.0	72.2	60.0	65.5	
Yes	2.9	2.8	8.7	4.3		24.6	29.0	33.0	33.3		6.6	9.7	15.1	10.0	
Unknown	8.3	11.6	23.9	13.0		4.8	5.7	4.7	6.0		15.3	18.2	24.9	24.5	
Lymph node extension					<0.001					0.001					<0.001
None	75.8	75.6	63.0	69.6		68.0	70.3	69.3	60.7		71.6	66.6	49.4	54.1	
Regional	0.7	1.2	6.5	4.3		14.9	20.0	11.6	23.2		4.8	8.6	14.1	9.3	
Distant	0.1	0	2.2	1.4		0.9	41.3	1.4	0		0.8	1.2	2.7	2.3	
Unknown	23.4	23.2	28.3	24.6		16.2	8.3	17.7	16.1		22.8	23.6	33.9	34.3	
Stage					<0.001					0.005					<0.001
In situ	-	-	-	-		-	-	-	-		-	-	-	-	
Localized	93.5	93.2	73.9	85.5		67.7	60.7	66.5	53.0		77.3	66.3	44.7	57.7	
Regional	3.2	3.2	17.4	8.7		27.2	31.3	26.5	39.9		8.8	14.6	21.1	15.3	
Distant	0.3	0.4	4.3	1.4		3.3	4.3	4.7	5.4		6.3	10.3	20.6	16.4	
Unstaged	2.9	3.2	4.3	4.3		1.8	3.7	2.3	1.8		7.7	8.8	13.7	10.6	
Treatment					0.016					0.028					<0.001
No treatment	2.1	4.0	4.3	1.4		1.8	2.7	3.3	0.6		6.1	11.2	15.4	12.1	
Surgery	96.2	94.8	93.5	92.8		96.8	94.3	94.4	94.6		88.7	82.5	73.0	78.0	
Radiation	0.1	0	0	1.4		0.2	0.3	0	0.6		1.7	2.0	4.8	3.9	
Radiation & Surgery	0.9	0.4	0	2.9		1.3	2.3	2.3	3.6		2.5	3.6	4.1	4.4	
Unknown	0.6	0.8	2.2	1.4		0	0.3	0	0.6		1.1	0.7	2.8	1.5	
Survival time (month)	181.9 ± 2.0	188.7 ± 14.2	153.0 ± 21.9	237.3 ± 25.1	0.138	158.4 ± 4.0	154.4 ± 10.4	133.4 ± 9.9	130.1 ± 12.1	0.023	243.0 ± 0.9	237.9 ± 5.8	144.6 ± 8.9	215.3 ± 10.1	<0.001

Cancers diagnosed 1973-2011 in the SEER 18 database. For a continuous variable, mean ± standard deviation; For a categorical variable, percentage

### Statistical analysis

Data on the six subtypes were analyzed separately. In the analysis of patients’ characteristics, Chi-squared tests and ANOVA were used to compare across racial groups for categorical and continuous variables respectively. Age-adjusted incidence rates were computed using SEER*Stat and the U.S. Census 2000 data for age-standardization. Five-year survival rates were calculated using SEER*Stat and an actuarial method. Treatment was analyzed using multivariate logistic regression, adjusted for age at diagnosis, gender, marital status, anatomic site, thickness of tumor, and ulceration. Survival was analyzed using multivariate Cox regression, adjusted for age at diagnosis, gender, marital status, anatomic site, thickness of tumor, ulceration, and treatment. Analysis not achievable using SEER software was conducted using SAS 9.3.

## Results

### Patients’ clinicopathologic characteristics

Results are shown in Table 1. Data on 376,797 patients are analyzed. For all variables of interest, significant racial differences are observed for multiple or all subtypes, and the patterns vary across subtypes. Specifically, for MIS, there are more male patients for NHW but more females for other races (*p*-value < 0.001). For NS, there are more male patients for NHW and HW but not the other two races. For LMM, there are more male patients across all races, although the percentages differ (*p*-value < 0.001). Significant racial differences in marital status are observed for all subtypes expect for LMM. Blacks have consistently lower rates of being married. Age at diagnosis significantly differs across races for all subtypes. For MIS and LMM, Asians/PIs and Blacks have the lowest age at diagnosis. For the other four subtypes, HWs have the lowest. For all subtypes, there are significant racial differences in anatomic site, and the patterns differ across subtypes. For example for MIS, the most prevalent are trunk (NHW, 30.9 %), face/head/neck (HW, 28.6 %), lower extremity (Black, 44.1 %), and lower extremity (Asian/PI, 29.0 %), respectively. For ALM, lower extremity is the dominating category for all races, although the percentages differ. For MIS, there is no racial difference in thickness of tumor. For NM and ALM, Asians/PIs have the thickest tumors, whereas for the other three subtypes, Blacks have the thickest. For the five subtypes with satellite nodule definition, the dominating majority of patients have no satellite nodule. The percentages differ significantly across races except for NOS. The distribution of skin ulceration differs significantly across races for all subtypes except for MIS, but the patterns differ across subtypes. For example, for SMM, the percentages of “No Ulceration” are 86.7, 87.6, 83.5, and 87.4 % for the four races, whereas for NOS, the corresponding percentages are 78.0, 72.2, 60.0, and 65.5 %, respectively. For MIS, almost all patients have no lymph node extension, and thus there is no racial difference. For the other five subtypes, there are significant racial differences in lymph node extension. For SMM, there are more HWs without lymph node extension, whereas there are more NHWs for NM. Except for MIS, significant racial differences are observed in stage, with more NHWs having localized tumors. The dominating majority of patients were treated with surgery, with significant racial differences except for MIS. For all subtypes except for LMM, there are significant racial differences in survival time. The racial groups that have the longest survival are HW for MIS and SSM and NHW for NM, ALM, and NOS.

### Incidence

Results are shown in Table 2. The sample sizes are 49,313 (MIS), 46,860 (SSM), 9,639 (NM), 9,912 (LMM), 1,506 (ALM), and 62,622 (NOS), respectively. For the six subtypes, the overall incidence rates per 100,000 person-years are 6.60, 6.18, 1.30, 1.37, 0.20, and 8.36, respectively. For all subtypes except for ALM, NHWs have the highest age-adjusted incidence rates, followed by HWs, while Blacks have the lowest. In the stratified analysis by age and gender, the same pattern holds. In addition, it is observed that incidence increases with age. For most cases, males have higher incidence, with exceptions including HWs with MIS and SSM. For ALM overall, HWs have the highest incidence rate (0.24), followed by NHWs (0.21), while Asians/PIs have the lowest rate (0.17). In the stratified analysis by age, NHWs have the highest rate for the <40 years age group, while HWs have the highest rates for the 40–64 and 65+ years groups. When stratified by gender, HWs have the highest rates for both groups. The incidence of ALM also increases with age. The incidence rates are similar for male and female.

**Table 2:** Age-adjusted incidence rates per 100,000 person-years, stratified by age and gender

	NHW	HW	Black	Asian/PI	Total
Melanoma in situ					
All ages	9.19 (9.11–9.28)	1.26 (1.18–1.35)	0.16 (0.13–0.20)	0.34 (0.30–0.38)	6.60 (6.54–6.66)
<40 years	2.18 (2.12–2.24)	0.21 (0.18–0.24)	0.03 (0.02–0.05)	0.10 (0.08–0.13)	1.31 (1.27–1.34)
40–64 years	13.97 (13.78–14.16)	1.61 (1.47–1.76)	0.19 (0.14–0.26)	0.48 (0.40–0.57)	9.82 (9.69–9.94)
65+ years	29.35 (28.93–29.77)	5.14 (4.63–5.70)	0.67 (0.49–0.90)	1.07 (0.87–1.31)	22.72 (22.41–23.04)
Male	10.71 (10.58–10.85)	1.22 (1.09–1.35)	0.19 (0.14–0.26)	0.35 (0.29–0.42)	7.95 (7.85–8.05)
Female	8.33 (8.22–8.45)	1.37 (1.26–1.48)	0.15 (0.11–0.19)	0.34 (0.29–0.39)	5.77 (5.70–5.85)
Superficial spreading melanoma					
All ages	9.05 (8.96–9.13)	1.12 (1.05–1.19)	0.15 (0.12–0.18)	0.31 (0.27–0.35)	6.18 (6.13–6.24)
<40 years	3.35 (3.27–3.42)	0.35 (0.32–0.39)	0.04 (0.02–0.06)	0.11 (0.08–0.14)	1.96 (1.92–2.00)
40–64 years	14.73 (14.54–14.93)	1.81 (1.66–1.97)	0.20 (0.15–0.27)	0.47 (0.39–0.56)	10.04 (9.91–10.17)
65+ years	21.09 (20.74–21.45)	2.91 (2.53–3.33)	0.51 (0.35–0.71)	0.81 (0.64–1.02	15.93 (15.67–16.20)
Male	10.23 (10.10–10.37)	0.98 (0.88–1.09)	0.18 (0.13–0.24)	0.34 (0.28–0.41)	7.20 (7.11–7.29)
Female	8.27 (8.16–8.39)	1.29 (1.19–1.39)	0.13 (0.09–0.17)	0.29 (0.24–0.34)	5.49 (5.42–5.57)
Nodular melanoma					
All ages	1.80 (1.76–1.84)	0.49 (0.44–0.54)	0.06 (0.04–0.08)	0.14 (0.12–0.17)	1.30 (1.28–1.33)
<40 years	0.38 (0.35–0.40)	0.08 (0.06–0.10)	0.01 (0.00–0.02)	0.04 (0.02–0.06)	0.23 (0.21–0.24)
40–64 years	2.31 (2.24–2.39)	0.51 (0.43–0.60)	0.06 (0.03–0.10)	0.14 (0.10–0.19)	1.60 (1.55–1.65)
65+ years	6.98 (6.78–7.19)	2.26 (1.92–2.65)	0.31 (0.19–0.48)	0.63 (0.47–0.82)	5.46 (5.30–5.61)
Male	2.51 (2.45–2.58)	0.60 (0.51–0.70)	0.07 (0.04–0.12)	0.19 (0.14–0.24)	1.84 (1.80–1.89)
Female	1.26 (1.22–1.30)	0.42 (0.36–0.48)	0.05 (0.03–0.08)	0.11 (0.08–0.15)	0.91 (0.88–0.93)
Lentigo maligna melanoma					
All ages	1.87 (1.83–1.90)	0.23 (0.19–0.27)	0.02 (0.01–0.04)	0.06 (0.05–0.08)	1.37 (1.35–1.40)
<40 years	0.05 (0.04–0.06)	0.01 (0.00–0.01)	0.00	0.01 (0.00–0.02)	0.03 (0.03–0.04)
40–64 years	1.83 (1.77–1.90)	0.17 (0.13–0.23)	0.01 (0.00–0.04)	0.05 (0.03–0.09)	1.26 (1.22–1.31)
65+ years	10.11 (9.87–10.36)	1.38 (1.11–1.69)	0.16 (0.08–0.29)	0.33 (0.22–0.47)	7.69 (7.51–7.87)
Male	2.97 (2.90–3.04)	0.33 (0.26–0.41)	0.04 (0.02–0.08)	0.09 (0.06–0.13)	2.21 (2.16–2.26)
Female	1.05 (1.02–1.09)	0.17 (0.13–0.21)	0.01 (0.00–0.03)	0.04 (0.02–0.06)	0.77 (0.74–0.80)
Acral lentiginous melanoma, malignant					
All ages	0.21 (0.20–0.22)	0.24 (0.21–0.28)	0.19 (0.16–0.23)	0.17 (0.14–0.20)	0.20 (0.19–0.22)
<40 years	0.04 (0.03–0.05)	0.02 (0.01–0.02)	0.02 (0.01–0.03)	0.02 (0.01–0.03)	0.03 (0.03–0.04)
40–64 years	0.25 (0.23–0.28)	0.26 (0.20–0.32)	0.21 (0.16–0.28)	0.17 (0.13–0.23)	0.24 (0.22–0.26)
65+ years	0.87 (0.80–0.95)	1.24 (0.99–1.54)	0.93 (0.71–1.20)	0.85 (0.67–1.07)	0.90 (0.84–0.96)
Male	0.22 (0.20–0.24)	0.25 (0.19–0.31)	0.22 (0.16–0.30)	0.20 (0.16–0.26)	0.22 (0.20–0.23)
Female	0.21 (0.19–0.23)	0.24 (0.20–0.30)	0.18 (0.14–0.22)	0.14 (0.11–0.18)	0.20 (0.18–0.21)
Malignant melanoma, NOS					
All ages	11.73 (11.64–11.83)	2.25 (2.14–2.36)	0.51 (0.45–0.57)	0.66 (0.60–0.72)	8.36 (8.29–8.42)
<40 years	3.37 (3.30–3.44)	0.48 (0.44–0.53)	0.10 (0.07–0.13)	0.17 (0.14–0.21)	2.02 (1.98–2.07)
40–64 years	17.35 (17.14–17.56)	2.98 (2.78–3.18)	0.57 (0.48–0.67)	0.83 (0.72–0.94)	12.10 (11.96–12.24)
65+ years	35.91 (35.45–36.38)	8.46 (7.79–9.18)	2.22 (1.87–2.62)	2.42 (2.11–2.77)	27.92 (27.58–28.28)
Male	14.63 (14.47–14.79)	2.31 (2.13–2.49)	0.63 (0.53–0.75)	0.69 (0.61–0.79)	10.68 (10.57–10.80)
Female	9.70 (9.58–9.82)	2.30 (2.16–2.44)	0.43 (0.36–0.50)	0.64 (0.57–0.72)	6.74 (6.66–6.82)

Diagnosed in the period of 1992–2011 in the SEER 13 database. In each cell, estimate (95 % CI). Rates are age-standardized using the U.S. 2000 Census population

### Treatment

The analysis is conducted on 90,183 (MIS), 85,813 (SSM), 19,779 (NM), 16,987 (LMM), 2,454 (ALM), and 122,314 (NOS) samples, and summary results are shown in Table 3. Detailed logistic regression analysis results are available from the authors. For the first five subtypes and localized and regional NOS, the dominating majority of patients had surgery. For distant NOS, the distribution of treatment is: 38.0 % no surgery or radiation, 27.6 % surgery, 15.2 % radiation, and 19.1 % both radiation and surgery. In the multivariate logistic regression, there are no significant racial differences for the first four subtypes and localized and regional ALM. For the distant stage of ALM, the multivariate logistic regression generates a significant *p*-value (0.022). It is noted that this significance should be interpreted with cautions because of the small counts. For localized and regional NOS, significant differences are observed across races. For localized, NHWs had the highest rate of surgery (97.4 %), while Blacks had the lowest (94.6 %). For regional, Blacks had the highest rate of surgery (92.2 %), while HWs had the lowest (81.7 %). For distant, there are many more patients in the “no surgery or radiation treatment” category, and there is significant difference across races (*p*-value < 0.001).

**Table 3:** Treatment strategy, stratified by stage-at-diagnosis

		NHW	HW	Black	Asian/PI	*P*-value
Melanoma in situ						
In situ	No treatment	3641 (4.2)	109 (5.8)	11 (5.0)	17 (4.2)	0.142
	Surgery	83560 (95.3)	1770 (93.8)	210 (95.0)	385 (95.5)	
	Radiation	117 (0.1)	2 (0.1)	0	1 (0.2)	
	Radiation & Surgery	354 (0.4)	6 (0.3)	0	0	
Superficial spreading melanoma						
Localized	No treatment	1106 (1.4)	38 (2.1)	3 (1.6)	8 (2.5)	0.877
	Surgery	76782 (98.0)	1764 (97.5)	183 (97.9)	306 (95.6)	
	Radiation	16 (0.0)	0	0	0	
	Radiation & Surgery	440 (0.6)	7 (0.4)	1 (0.5)	6 (1.9)	
Regional	No treatment	19 (0.4)	0	0	0	0.999
	Surgery	4366 (95.4)	142 (96.6)	20 (95.2)	34 (97.1)	
	Radiation	4 (0.1)	0	0	0	
	Radiation & Surgery	188 (4.1)	5 (3.4)	1 (4.8)	1 (2.9)	
Distant	No treatment	15 (4.2)	0	0	0	0.650
	Surgery	259 (73.4)	12 (80.0)	1 (50.0)	3 (100.0)	
	Radiation	9 (2.5)	0	0	0	
	Radiation & Surgery	70 (19.8)	3 (20.0)	1 (50.0)	0	
Nodular melanoma						
Localized	No treatment	149 (1.3)	5 (1.4)	2 (4.5)	1 (1.2)	0.585
	Surgery	11613 (97.5)	344 (98.0)	42 (95.5)	81 (97.6)	
	Radiation	12 (0.1)	0	0	1 (1.2)	
	Radiation & Surgery	137 (1.2)	2 (0.6)	0	0	
Regional	No treatment	41 (0.7)	2 (0.7)	0	0	0.571
	Surgery	5792 (94.2)	259 (93.8)	39 (95.1)	63 (95.5)	
	Radiation	2 (0.0)	0	0	1 (1.5)	
	Radiation & Surgery	312 (5.1)	15 (5.4)	2 (4.9)	2 (3.0)	
Distant	No treatment	42 (5.3)	4 (8.2)	0	0	0.346
	Surgery	553 (70.3)	40 (81.6)	11 (73.3)	6 (66.7)	
	Radiation	9 (1.1)	0	0	0	
	Radiation & Surgery	183 (23.3)	5 (10.2)	4 (26.7)	3 (33.3)	
Lentigo maligna melanoma						
Localized	No treatment	301 (1.9)	7 (3.0)	1 (2.9)	0	0.986
	Surgery	15617 (97.4)	224 (96.6)	33 (97.1)	56 (96.6)	
	Radiation	5 (0.0)	0	0	1 (1.7)	
	Radiation & Surgery	104 (0.6)	1 (0.4)	0	1 (1.7)	
Regional	No treatment	4 (0.7)	0	1 (12.5)	0	0.759
	Surgery	513 (92.9)	8 (100.0)	7 (87.5)	5 (83.3)	
	Radiation	-	-	-	-	
	Radiation & Surgery	35 (6.3)	0	0	1 (16.7)	
Distant	No treatment	2 (3.4)	0	0	0	0.992
	Surgery	40 (69.0)	1 (100.0)	2 (100.0)	1 (100.0)	
	Radiation	2 (3.4)	0	0	0	
	Radiation & Surgery	14 (24.1)	0	0	0	
Acral lentiginous melanoma, malignant						
Localized	No treatment	23 (1.9)	3 (1.6)	5 (3.5)	1 (1.1)	0.841
	Surgery	1201 (97.3)	178 (97.8)	138 (96.5)	87 (97.8)	
	Radiation	2 (0.2)	0	0	0	
	Radiation & Surgery	8 (0.6)	1 (0.5)	1 (0.5)	1 (1.1)	
Regional	No treatment	3 (0.6)	0	1 (1.8)	0	0.301
	Surgery	481 (97.2)	89 (94.7)	54 (94.7)	65 (97.0)	
	Radiation	-	-	-	-	
	Radiation & Surgery	11 (2.2)	5 (5.3)	2 (3.5)	2 (3.0)	
Distant	No treatment	1 (1.6)	1 (7.7)	0	0	0.022
	Surgery	54 (88.5)	11 (84.6)	7 (70.0)	5 (55.6)	
	Radiation	1 (1.6)	1 (7.7)	0	1 (11.1)	
	Radiation & Surgery	5 (8.2)	0	3 (33.3)	3 (30.0)	
Malignant melanoma, NOS						
Localized	No treatment	1950 (2.0)	84 (3.3)	14 (4.0)	18 (3.6)	<0.001
	Surgery	95929 (97.4)	2425 (96.2)	330 (94.6)	475 (95.6)	
	Radiation	70 (0.1)	0	2 (0.6)	0	
	Radiation & Surgery	591 (0.6)	13 (0.5)	3 (0.9)	4 (0.8)	
Regional	No treatment	882 (7.9)	52 (9.4)	2 (1.2)	7 (5.3)	0.010
	Surgery	9397 (84.3)	451 (81.7)	153 (92.2)	116 (87.9)	
	Radiation	69 (0.6)	7 (1.3)	1 (0.6)	1 (0.8)	
	Radiation & Surgery	801 (7.2)	42 (7.6)	10 (6.0)	8 (6.1)	
Distant	No treatment	2901 (37.5)	175 (44.9)	71 (46.1)	50 (37.6)	<0.001
	Surgery	2136 (27.6)	97 (24.9)	51 (33.1)	40 (30.1)	
	Radiation	1192 (15.4)	48 (12.3)	17 (11.0)	25 (18.8)	
	Radiation & Surgery	1501 (19.4)	70 (17.9)	15 (9.7)	18 (13.5)	

Cancers diagnosed in the period of 1973–2011 in the SEER 18 database. In each cell, count (percentage). *P*-values were obtained from multivariate logistic regression

### Survival

**Fig. 1:**
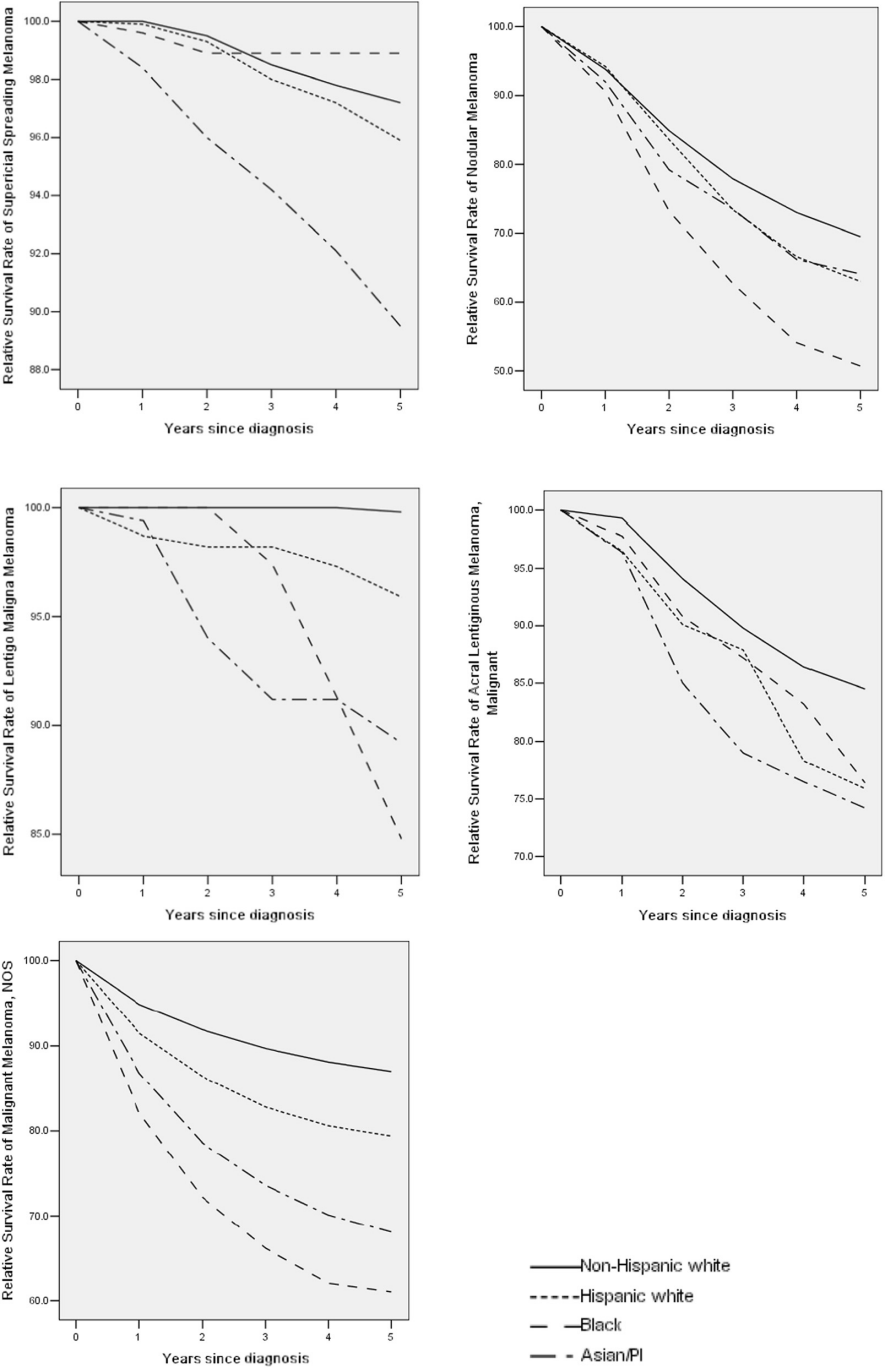
Relative survival rates up to five years, for all stages combined. Cancers diagnosed in the period of 1973–2006 and followed up to 12/31/2011

**Fig. 2:**
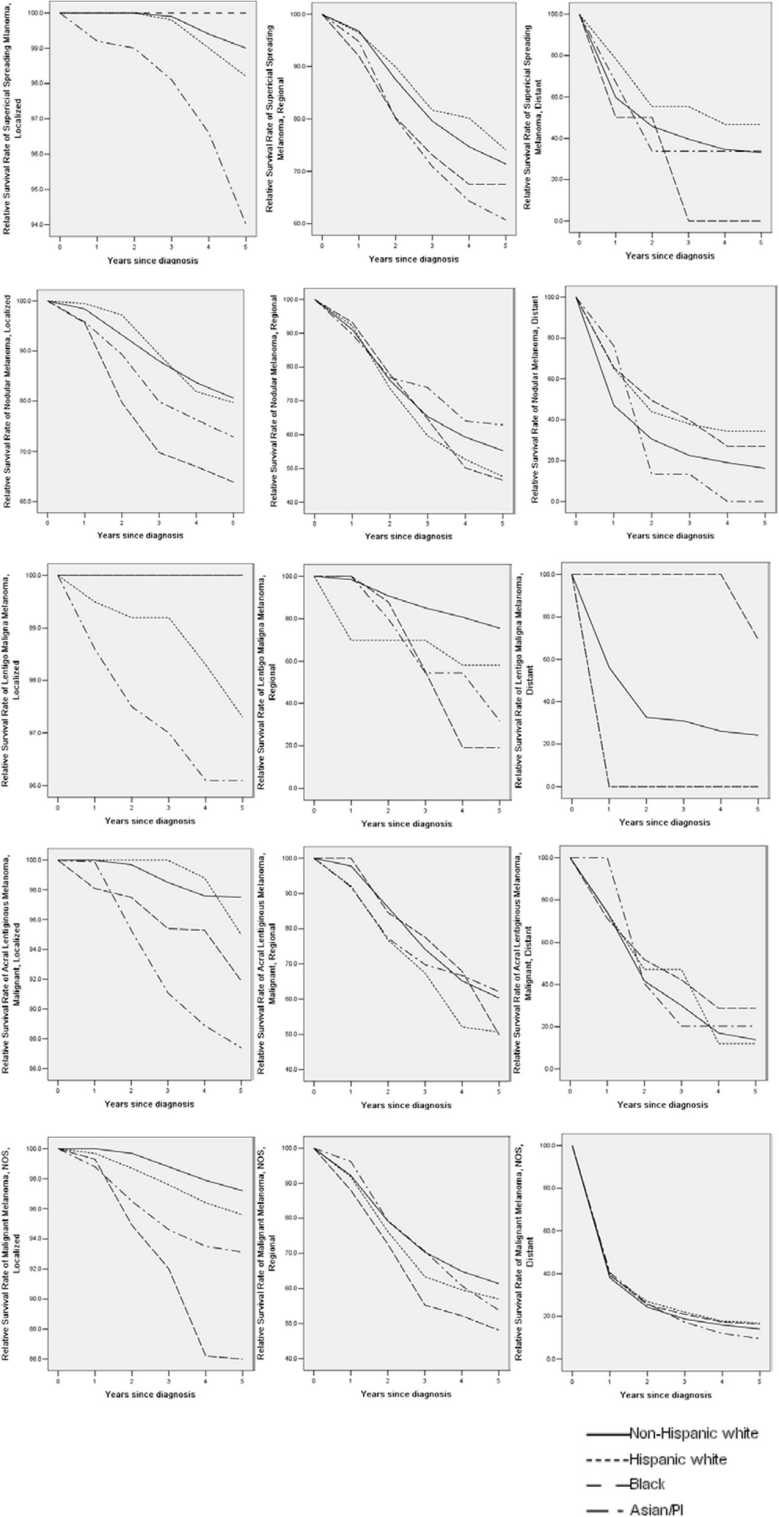
Relative survival rates up to five years, stratified by stage. Cancers diagnosed in the period of 1973–2006 and followed up to 12/31/2011

The analysis is based on 70,898 (MIS), 74,490 (SSM), 16,286 (NM), 12,507 (LMM), 2,047 (ALM), and 100,865 (NOS) samples. The summary results are shown in Table 4. Detailed multivariate Cox regression analysis results are shown in Table 5 in Appendix. The survival curves for up to five years are shown in Fig. 1 (all stages combined) and Fig. 2 (stratified by stage at diagnosis). Note that for MIS, the five-year survival rates are 100 % and thus not plotted. For SSM, the racial groups with the best five-year survival are Black (localized, 100 %), HW (regional, 74.1 %), and HW (distant, 46.8 %). For NS, the racial groups with the best survival are NHW (localized, 80.6 %), Asian/PI (regional, 62.9 %), and HW (distant, 34.4 %). For LMM, the groups with the best survival are Black and NHW (localized, 100 %), NHW (regional, 75.6 %), and Black (distant, 69.4 %). For ALM, the groups with the best survival are NHW (localized, 97.5 %), Asian/PI (regional, 62.1 %), and Black (distant, 28.7 %). For NOS, NHWs have the best survival with localized (97.2 %) and regional (61.4 %) tumors, and HWs have the best survival with distant tumors (17.0 %). For the first five subtypes, racial differences are not significant in the Cox regression after accounting for confounders. For NOS, significant racial differences are observed for the localized and regional stages. Figures 1 and 2 provide more detailed information on the survival rates between time zero and year five. Figure 2 shows that the localized stage has the best relative survival rates for all five subtypes. In contrast, the distant stage has the worst survival rates. The separation of survival curves is the most distinct for SMM with the localized stage, LMM with the localized and distant stages, and NOS with the distant stage, while there are some crossovers for the other subtypes.

**Table 4:** Five-year relative survival rates, stratified by stage-at-diagnosis

	Total	NHW	HW	Black	Asian/PI	*P*-value
Melanoma in situ						
In situ	100.0	100.0	100.0	100.0	100.0	0.380
Superficial spreading melanoma						
Localized	99.2 (98.9–99.4)	99.0 (98.7–99.2)	98.2 (96.1–99.2)	100.0	94.0 (87.7–97.1)	0.844
Regional	71.5 (69.7–73.2)	71.4 (69.6–73.2)	74.1 (62.9–82.4)	67.5 (33.5–86.8)	60.7 (40.0–76.3)	0.623
Distant	33.8 (27.7–40.0)	33.0 (26.7–39.5)	46.8 (18.1–71.4)	0.0	33.7 (0.9–77.9)	0.987
Nodular melanoma						
Localized	80.6 (79.5–81.7)	80.6 (79.4–81.7)	79.7 (72.4–85.4)	63.9 (39.0–80.8)	72.9 (56.8–83.8)	0.403
Regional	55.1 (53.4–56.7)	55.3 (53.6–57.0)	47.6 (39.5–55.1)	46.5 (24.7–65.7)	62.9 (46.9–75.3)	0.119
Distant	17.1 (13.9–20.6)	16.2 (12.9–19.7)	34.4 (17.6–51.9)	27.1 (4.9–56.8)	13.3 (0.7–44.3)	0.101
Lentigo maligna melanoma						
Localized	100.0	100.0	97.3 (61.0–99.8)	100.0	96.1 (53.3–99.8)	0.697
Regional	73.2 (65.6–79.4)	75.6 (67.7–81.8)	58.1 (3.7–91.5)	19.1 (0.8–56.9)	31.7 (0.7–76.9)	0.151
Distant	26.1 (11.3–43.8)	24.2 (9.5–42.4)	0.0	69.4 (0.0–99.2)	0.0	0.508
Acral lentiginous melanoma, malignant						
Localized	96.0 (92.6–97.8)	97.5 (92.4–99.2)	95.0 (75.3–99.1)	91.9 (74.9–97.6)	87.4 (70.7–94.9)	0.266
Regional	58.4 (52.9–63.5)	60.3 (53.5–66.5)	50.6 (37.0–62.7)	49.8 (29.3–67.3)	62.1 (44.5–75.5)	0.447
Distant	15.8 (7.7–26.5)	13.8 (4.8–27.5)	12.0 (0.7–40.7)	28.7 (4.9–59.7)	20.3 (0.8–58.8)	0.651
Malignant melanoma, NOS						
Localized	97.3 (97.1–97.6)	97.2 (96.9–97.5)	95.6 (93.7–96.9)	86.0 (78.2–91.1)	93.1 (88.6–95.9)	0.013
Regional	61.1 (59.8–62.2)	61.4 (60.2–62.7)	57.0 (51.4–62.2)	48.1 (37.7–57.7)	53.8 (42.8–63.6)	0.007
Distant	14.3 (13.4–15.3)	14.1 (13.1–15.1)	17.0 (12.5–22.1)	16.4 (9.7–24.6)	9.6 (4.6–16.7)	0.501

Cancers diagnosed in the period of 1973–2006 and followed up to 12/31/2011 in the SEER 18 database. In each cell, estimated rate (95 % CI). *P*-values were obtained from multivariate Cox regression

**Table 5:** Multivariate Cox regression analysis of survival, stratified by stage at diagnosis

	In situ			Localized			Regional			Distant		
	HR	95 % CI	*P*	HR	95 % CI	*P*	HR	95 % CI	*P*	HR	95 % CI	*P*
Melanoma in situ												
Gender				-	-	-	-	-	-	-	-	-
Male	1			-	-	-	-	-	-	-	-	-
Female	0.502	0.398–0.634	0.000	-	-	-	-	-	-	-	-	-
Age at diagnosis	1.098	1.087–1.110	0.000	-	-	-	-	-	-	-	-	-
Marital Status				-	-	-	-	-	-	-	-	-
Single	1			-	-	-	-	-	-	-	-	-
Married	0.770	0.539–1.099	0.150	-	-	-	-	-	-	-	-	-
Separated/divorced/widowed	1.225	0.831–1.806	0.305	-	-	-	-	-	-	-	-	-
Ethnic group				-	-	-	-	-	-	-	-	-
NHW	1			-	-	-	-	-	-	-	-	-
HW	1.886	0.888–4.006	0.099	-	-	-	-	-	-	-	-	-
Black	0.709	0.098–5.115	0.733	-	-	-	-	-	-	-	-	-
Asian/PI	0.624	0.087–4.495	0.640	-	-	-	-	-	-	-	-	-
Anatomic Site				-	-	-	-	-	-	-	-	-
Face/head/neck	1			-	-	-	-	-	-	-	-	-
Trunk	0.993	0.783–1.259	0.952	-	-	-	-	-	-	-	-	-
Upper Extremity	0.876	0.668–1.149	0.338	-	-	-	-	-	-	-	-	-
Lower Extremity	0.753	0.500–1.133	0.174	-	-	-	-	-	-	-	-	-
Other	1.081	0.399–2.929	0.878	-	-	-	-	-	-	-	-	-
Treatment				-	-	-	-	-	-	-	-	-
No surgery or radiation	1			-	-	-	-	-	-	-	-	-
Surgery	1.125	0.531–2.387	0.758	-	-	-	-	-	-	-	-	-
Radiation	0.185	--	0.989	-	-	-	-	-	-	-	-	-
Radiation & Surgery	1.041	0.214–5.063	0.961	-	-	-	-	-	-	-	-	-
Thickness of tumor (mm)	1.043	0.993–1.095	0.091	-	-	-	-	-	-	-	-	-
Ulceration				-	-	-	-	-	-	-	-	-
No	1			-	-	-	-	-	-	-	-	-
Yes	1.722	0.847–3.503	0.133	-	-	-	-	-	-	-	-	-
Superficial spreading melanoma												
Gender												
Male	-	-	-	1			1			1		
Female	-	-	-	0.709	0.677–0.742	0.000	0.718	0.644–0.801	0.000	0.657	0.452–0.955	0.028
Age at diagnosis	-	-	-	1.074	1.072–1.075	0.000	1.033	1.029–1.036	0.000	1.018	1.007–1.029	0.001
Marital Status												
Single	-	-	-	1			1			1		
Married	-	-	-	0.741	0.693–0.792	0.000	0.724	0.631–0.832	0.000	0.505	0.337–0.757	0.001
Separated/divorced/widowed	-	-	-	1.034	0.957–1.117	0.400	0.897	0.759–1.059	0.199	0.791	0.480–1.301	0.355
Ethnic group												
NHW	-	-	-	1			1			1		
HW	-	-	-	1.065	0.915–1.241	0.416	1.213	0.909–1.620	0.190	0.931	0.468–1.850	0.837
Black	-	-	-	0.920	0.605–1.398	0.696	0.954	0.549–1.657	0.867	1.171	0.156–8.812	0.878
Asian/PI	-	-	-	1.008	0.726–1.398	0.964	1.028	0.606–1.745	0.918	0.821	0.196–3.447	0.788
Anatomic Site												
Face/head/neck	-	-	-	1			1			1		
Trunk	-	-	-	0.849	0.804–0.896	0.000	0.896	0.788–1.020	0.098	1.570	1.062–2.319	0.024
Upper Extremity	-	-	-	0.750	0.707–0.795	0.000	0.800	0.692–0.925	0.003	1.606	1.034–2.493	0.035
Lower Extremity	-	-	-	0.655	0.611–0.701	0.000	0.820	0.707–0.951	0.009	1.384	0.825–2.323	0.218
Other	-	-	-	1.031	0.731–1.455	0.860	1.359	0.507–3.646	0.542	0.000	--	0.954
Treatment												
No surgery or radiation	-	-	-	1			1			1		
Surgery	-	-	-	0.584	0.468–0.729	0.000	2.030	0.652–6.316	0.222	0.274	0.116–0.645	0.003
Radiation	-	-	-	1.738	0.427–7.077	0.440	5.760	0.597–55.57	0.130	1.454	0.397–5.328	0.572
Radiation & Surgery	-	-	-	1.175	0.841–1.640	0.345	4.154	1.316–13.11	0.015	0.750	0.306–1.836	0.529
Thickness of tumor (mm)	-	-	-	1.223	1.207–1.239	0.000	1.152	1.128–1.176	0.000	1.107	1.043–1.175	0.001
Ulceration												
No	-	-	-	1			1			1		
Yes	-	-	-	1.355	1.226–1.498	0.000	1.082	0.984–1.190	0.102	1.011	0.703–1.453	0.954
Nodular melanoma												
Gender												
Male	-	-	-	1			1			1		
Female	-	-	-	0.753	0.698–0.812	0.000	0.790	0.728–0.858	0.000	1.069	0.879–1.299	0.504
Age at diagnosis	-	-	-	1.048	1.045–1.050	0.000	1.028	1.026–1.031	0.000	1.010	1.004–1.015	0.001
Marital Status												
Single	-	-	-	1			1			1		
Married	-	-	-	0.707	0.636–0.785	0.000	0.799	0.722–0.885	0.000	0.763	0.600–0.970	0.027
Separated/divorced/widowed	-	-	-	0.944	0.838–1.063	0.341	1.001	0.887–1.129	0.987	0.880	0.663–1.167	0.374
Ethnic group												
NHW	-	-	-	1			1			1		
HW	-	-	-	0.924	0.739–1.155	0.486	1.150	0.958–1.380	0.134	0.625	0.416–0.940	0.024
Black	-	-	-	1.533	0.887–2.647	0.126	1.335	0.876–2.035	0.179	0.597	0.243–1.467	0.261
Asian/PI	-	-	-	0.938	0.597–1.475	0.783	0.781	0.540–1.128	0.187	0.959	0.469–1.961	0.910
Anatomic Site												
Face/head/neck	-	-	-	1			1			1		
Trunk	-	-	-	1.046	0.957–1.143	0.320	1.125	1.023–1.238	0.016	1.284	1.027–1.605	0.029
Upper Extremity	-	-	-	0.846	0.773–0.926	0.000	0.901	0.813–0.998	0.046	0.960	0.745–1.237	0.750
Lower Extremity	-	-	-	0.832	0.743–0.931	0.001	0.984	0.879–1.101	0.775	0.936	0.711–1.232	0.636
Other	-	-	-	0.974	0.572–1.660	0.923	0.940	0.518–1.705	0.838	3.880	0.942–15.986	0.061
Treatment												
No surgery or radiation	-	-	-	1			1			1		
Surgery	-	-	-	0.498	0.358–0.692	0.000	0.416	0.276–0.628	0.000	0.459	0.282–0.748	0.002
Radiation	-	-	-	1.813	0.644–5.102	0.260	0.418	0.056–3.102	0.394	0.626	0.210–1.866	0.400
Radiation & Surgery	-	-	-	1.012	0.667–1.536	1.012	0.648	0.419–1.003	0.051	0.678	0.409–1.124	0.132
Thickness of tumor (mm)	-	-	-	1.120	1.104–1.136	0.000	1.074	1.060–1.088	0.000	1.017	0.989–1.044	0.235
Ulceration												
No	-	-	-	1			1			1		
Yes	-	-	-	1.215	1.116–1.323	0.000	1.074	1.000–1.155	0.051	0.834	0.699–0.997	0.046
Lentigo maligna melanoma												
Gender												
Male	-	-	-	1			1			1		
Female	-	-	-	0.641	0.589–0.698	0.000	0.641	0.471–0.872	0.005	1.968	0.468–8.271	0.355
Age at diagnosis	-	-	-	1.093	1.088–1.097	0.000	1.050	1.036–1.065	0.000	1.016	0.965–1.070	0.550
Marital Status												
Single	-	-	-	1			1			1		
Married	-	-	-	0.758	0.660–0.870	0.000	0.858	0.508–1.449	0.566	1.701	0.345–8.396	0.514
Separated/divorced/widowed	-	-	-	0.939	0.808–1.091	0.409	1.137	0.657–1.969	0.646	0.744	0.082–6.771	0.793
Ethnic group												
NHW	-	-	-	1			1			1		
HW	-	-	-	0.981	0.723–1.330	0.900	0.619	0.196–1.954	0.414	-	-	-
Black	-	-	-	1.029	0.486–2.180	0.940	9.996	1.239–80.642	0.031	0.308	0.009–10.073	0.508
Asian	-	-	-	0.587	0.244–1.412	0.234	1.120	0.331–3.793	0.855	-	-	-
Anatomic Site												
Face/head/neck	-	-	-	1			1			1		
Trunk	-	-	-	0.909	0.812–1.018	0.099	1.068	0.666–1.713	0.785	5.787	1.173-28.562	0.031
Upper Extremity	-	-	-	0.888	0.806–0.978	0.016	0.988	0.656–1.488	0.954	1.564	0.145–16.844	0.712
Lower Extremity	-	-	-	0.802	0.655–0.981	0.032	0.670	0.363–1.234	0.198	-	-	-
Other	-	-	-	1.218	0.792–1.873	0.369	-	-	-	-	-	-
Treatment												
No surgery or radiation	-	-	-	1			1			-	-	-
Surgery	-	-	-	0.734	0.530–1.015	0.062	1.768	0.245–12.759	0.572	1		
Radiation	-	-	-	0.017	--	0.902	-	-	-	135.011	--	0.000
Radiation & Surgery	-	-	-	1.202	0.703–2.056	0.501	3.478	0.445–27.195	0.235	7.513	1.689–33.427	0.008
Thickness of tumor (mm)	-	-	-	1.130	1.098–1.162	0.000	1.094	1.030–1.161	0.003	1.142	0.893–1.460	0.289
Ulceration												
No	-	-	-	1			1			1		
Yes	-	-	-	1.243	0.971–1.592	0.085	1.242	0.949–1.626	0.114	2.834	0.458–17.545	0.263
Acral lentiginous melanoma, malignant												
Gender												
Male	-	-	-	1			1			1		
Female	-	-	-	0.810	0.649–1.011	0.062	0.595	0.471–0.752	0.000	0.828	0.449–1.529	0.546
Age at diagnosis	-	-	-	1.069	1.059–1.079	0.000	1.025	1.017–1.034	0.000	1.040	1.013–1.067	0.003
Marital Status												
Single	-	-	-	1			1			1		
Married	-	-	-	0.922	0.638–1.332	0.665	0.790	0.550–1.136	0.204	0.340	0.120–0.967	0.043
Separated/divorced/widowed	-	-	-	1.063	0.719–1.572	0.761	1.093	0.744–1.606	0.652	0.337	0.099–1.147	0.082
Ethnic group												
Non-Hispanic white	-	-	-	1			1			1		
Hispanic white	-	-	-	0.771	0.530–1.123	0.176	1.219	0.884–1.682	0.228	0.987	0.409–2.381	0.977
Black	-	-	-	1.112	0.803–1.540	0.523	1.080	0.751–1.553	0.678	0.601	0.254–1.420	0.246
Asian	-	-	-	1.307	0.826–2.068	0.254	0.843	0.568–1.250	0.396	0.630	0.183–2.176	0.465
Anatomic Site												
Face/head/neck	-	-	-	1			1			-	-	-
Trunk	-	-	-	0.845	0.280–2.552	0.765	0.770	0.123–4.845	0.781	1		
Upper Extremity	-	-	-	0.978	0.447–2.140	0.956	0.933	0.275–3.162	0.912	0.253	0.045–1.403	0.116
Lower Extremity	-	-	-	1.004	0.472–2.139	0.991	1.277	0.383–4.250	0.691	0.302	0.060–1.509	0.145
Other	-	-	-	0.913	0.111–7.533	0.933	0.011	--	0.962	-	-	-
Treatment												
No surgery or radiation	-	-	-	1			1			1		
Surgery	-	-	-	0.372	0.162–0.855	0.020	0.065	0.020–0.213	0.000	0.150	0.031–0.718	0.018
Radiation	-	-	-	0.431	0.049–3.766	0.446	-	-	-	0.946	0.112–7.994	0.959
Radiation & Surgery	-	-	-	0.151	0.018–1.271	0.082	0.179	0.047–0.687	0.012	0.528	0.091–3.061	0.476
Thickness of tumor (mm)	-	-	-	1.137	1.072–1.206	0.000	1.115	1.069–1.163	0.000	1.094	0.988–1.211	0.083
Ulceration												
No	-	-	-	1			1			1		
Yes	-	-	-	1.544	1.112–2.143	0.009	1.103	0.888–1.368	0.376	2.281	1.216–4.280	0.010
Malignant melanoma, NOS												
Gender												
Male	-	-	-	1			1			1		
Female	-	-	-	0.705	0.676–0.735	0.000	0.800	0.742–0.862	0.000	0.881	0.752–1.032	0.117
Age at diagnosis	-	-	-	1.069	1.068–1.071	0.000	1.033	1.031–1.035	0.000	1.014	1.010–1.019	0.000
Marital Status												
Single	-	-	-	1			1			1		
Married	-	-	-	0.731	0.690–0.776	0.000	0.777	0.704–0.857	0.000	0.630	0.521–0.762	0.000
Separated/divorced/widowed	-	-	-	0.985	0.921–1.054	0.660	0.993	0.886–1.114	0.910	0.848	0.673–1.069	0.163
Ethnic group												
NHW	-	-	-	1			1			1		
HW	-	-	-	1.134	1.002–1.284	0.046	1.240	1.056–1.455	0.009	0.970	0.704–1.336	0.852
Black	-	-	-	1.389	1.086–1.777	0.009	1.372	1.056–1.782	0.018	0.859	0.516–1.431	0.561
Asian/PI	-	-	-	1.004	0.782–1.290	0.973	1.052	0.772–1.434	0.748	1.478	0.850–2.571	0.167
Anatomic Site												
Face/head/neck	-	-	-	1			1			1		
Trunk	-	-	-	0.918	0.876–0.962	0.000	1.087	0.994–1.189	0.068	1.098	0.913–1.320	0.320
Upper Extremity	-	-	-	0.811	0.772–0.852	0.000	0.859	0.779–0.948	0.003	0.970	0.782–1.203	0.778
Lower Extremity	-	-	-	0.732	0.687–0.779	0.000	0.910	0.823–1.006	0.065	0.835	0.672–1.037	0.103
Other	-	-	-	0.911	0.690–1.201	0.506	1.416	0.908–2.208	0.125	1.011	0.631–1.619	0.964
Treatment												
No surgery or radiation	-	-	-	1			1			1		
Surgery	-	-	-	0.656	0.572–0.752	0.000	0.392	0.291–0.530	0.000	0.472	0.357–0.626	0.000
Radiation	-	-	-	2.551	1.424–4.570	0.002	1.886	0.886–4.016	0.100	1.647	1.033–2.625	0.036
Radiation & Surgery	-	-	-	1.312	1.069–1.610	0.009	0.732	0.531–1.011	0.058	0.747	0.549–1.016	0.063
Thickness of tumor (mm)	-	-	-	1.196	1.183–1.208	0.000	1.086	1.073–1.099	0.000	1.045	1.021–1.069	0.000
Ulceration												
No	-	-	-	1			1			1		
Yes	-	-	-	1.448	1.351–1.553	0.000	1.172	1.097–1.252	0.000	1.002	0.858–1.172	0.975

Cancers diagnosed 1973–2006 and followed up to 12/31/2011

*HR* hazard ratio

## Discussion

### Main findings

The epidemiology of melanoma overall and its subtypes has been studied in a large number of publications. It has been noted that race causes differences in multiple aspects. However, most of the existing studies only include race as a confounding variable and have not paid sufficient attention. This study advances from the existing ones by comprehensively analyzing the six most major subtypes on the same ground for four largest racial groups. For the U.S. and other countries that have a significant race mixture, observations made in this study can provide valuable insights for public health and clinical investigators.

Melanoma is a rare disease in minorities. For all subtypes, there are dominatingly more NHW patients. The counts for the other races are relatively small for some subtypes. This can be especially problematic in the stratified analysis, and thus some results should be interpreted cautiously. For all of the patients’ characteristic variables examined in Table 1, significant racial differences are observed for at least some, if not all, subtypes, and the patterns vary across subtypes. The development of melanoma is extremely complicated. The heterogeneity in etiology and presentation among subtypes have been previously noted [14]. The observed differences across races and across subtypes reflect the complex interactions of occupational exposures (especially to UV light,) environment (closer to the equator or at a higher elevation), genetic makeup (which, for example, causes difference in skin and hair color), family history, deficiency in the immune system, and socioeconomic status. Some of the observed across-subtype differences, for example in gender and marital status, can be confounded with other factors such as socioeconomic status. In the literature [6, 15, 16], it has been suggested that minorities, especially Blacks and Hispanics, are more likely to be diagnosed at later stages and have advanced presentations. In our analysis, Blacks have stages later than the other races for all subtypes except for ALM. It has been suggested that this can be a consequence of economic, social, and cultural barriers such as low income, lack of insurance, lower levels of education, lower levels of melanoma awareness and knowledge, and lower rates of participation in melanoma screening [15, 17]. However in the literature, subtype-specific analysis of socioeconomic status is still lacking.

The incidence of melanoma is extremely complex. The most prominent risk factor is exposure to UV light. A large number of potential risk factors have been suggested, including presence of fair skin, freckling and light hair, family history, personal history of melanoma and other skin cancers, older age, male gender, and xeroderma pigmentosum (XP) [18]. In addition, a large number of genetic risk factors have been suggested, including germline mutations such as CDKN2A (which leads to destabilization of p53), CDK4, BAP1, MC1R, and MITF, and somatic mutations for example in the RAS-RAF-MEK-MAPK and PI3K-PTEN-AKT pathways [19]. For five out of six subtypes, the overall incidence rate patterns are relatively consistent across races. It has been long noted that NHWs have a higher incidence rate. This can be attributable to certain physical characteristics (for example light skin and hair color), lifestyle factors (for example more exposure to UV light), genetic risk factors, as well as others [20]. The analysis also suggests that there exists interaction between gender and race – it is observed that females have higher incidence among HWs for MIS and SSM, but for other cases, the trend is reversed. Previous studies have noted the interaction between gender and age for incidence [21]. However the interaction observed in this study has been less acknowledged. Some of the genetic risk factors may also interact with race. For example, all red-haired people have a mutated copy of MC1R. Overall, research on the distribution of genetic risk factors across races has been rare. The observation for ALM is different from the other subtypes. ALM is the most common type of melanoma in the Asian, Hispanic, and African populations. The fundamental difference of this subtype has been examined in the literature [22] and is not reiterated here.

The primary treatment for melanoma is surgical excision. Systemic adjuvant therapies (levamisole, interferon, vaccines, and chemotherapy) and radiotherapy may be considered for patients with high risk melanomas, including those with lymph node involvement and distant metastases. For most of the subtypes/stages, racial differences, although observed, are not statistically significant after adjusting for confounders. The difference observed for the distant stage of ALM should be taken with cautions because of the small sample size. For the localized stage of NOS, as most patients (at least 94.6 %) had surgery, the observed racial difference may not be clinically meaningful. For regional and distant tumors, the racial differences are more prominent. For regional tumors, Blacks had the highest rate of surgery (92.2 %), while HWs had the lowest (81.7 %). For distant tumors, all racial groups had more “scattered” treatment distributions. For a variety of solid tumors, racial differences in treatment have been well documented [23–25]. In particular, Blacks have been shown to be given less than optimal care [26, 27]. However, our analysis suggests that there is no such racial difference for melanoma treatment. A similar observation has been made for Blacks and Whites [28]. Treatment selection is a complex process involving multiple factors. The differences in patients’ characteristics, as previously observed, contribute to at least some of the differences. In addition, it has been suggested in the literature that socioeconomic status, insurance status, disparity (that is independent of socioeconomic status), cultural and behavioral differences all contribute to treatment selection [29]. It is noted that such information is not available from SEER.

Analysis suggests certain racial differences in the five-year survival rate. For example for localized NM, NHWs and Blacks have survival rates 80.6 and 63.9 %, respectively. However, after adjusting for confounders, racial differences are significant only for regional and distant NOS, with Blacks having the lowest survival rates. Multiple factors contribute to melanoma prognosis. Published studies have suggested potential prognostic roles of lesion thickness, ulceration, lymph node involvement, age, gender, anatomic site, satellite lesion, serum lactic dehydrogenase (LDH), and others. The differences in patients’ characteristics, as previously described, can contribute to survival difference. In addition, the aforementioned prognostic factors may also interact with race (for example the distribution of LDH varies across races [30]). Racial differences in survival have been examined in the literature. For example, Collins and others found that both overall and melanoma-specific survival was lower in Blacks undergoing surgical treatment compared to Whites and other races [5]. However, reasons for these differences remain poorly understood. Several possible explanations have been raised. For example, compared to Whites, Blacks were more likely to be diagnosed at more advanced stages [16, 31]. They were also more likely to have tumor ulceration, satellite nodules, and regional and distant metastases [3, 32–35]. Studies have also suggested that factors not measured in SEER, such as socioeconomic status, skin cancer awareness, and cultural and social values, may be related to racial differences in survival. Multiple genetic risk factors have also been suggested as having independent contributions to survival [36]. However, their interactions with race have not been examined.

### Limitations

The SEER database is analyzed as it is the most comprehensive cancer registry in the U.S. However, it has limitations. The most significant limitation is a lack of certain important measurements, such as UV exposure, socioeconomic status, lifestyle, and genetic risk factors. In addition, the treatment information is also not complete: there is no information on chemotherapy, biologic therapy, and others. Connecting to other databases or more data collection are needed. This study may also have been hindered by the multiple coexisting classification schemes. Patients diagnosed before 2001 may have diagnosis codes from earlier ICD-O versions that need to be converted to ICD-O-3, which may have resulted in unclassified cases. SEER has multiple sites, and errors may arise in tumor classification and staging. However, we do not expect systematic errors correlated with race. The SEER population have a higher proportion of foreign-born patients than the general U.S. population. Combined with the fact that SEER is limited to the U.S. only, there may be concerns on the generalizability of findings.

## Conclusions

This epidemiologic study has provided comprehensive descriptive statistics on racial differences in multiple aspects of major melanoma subtypes. Similar to many published studies of the same kind, it cannot reveal the underlying mechanisms that cause racial differences. However, it has been shown repeatedly in the literature that this kind of studies has extensive values. A major advancement of this study is its comprehensiveness: six subtypes are analyzed on the same ground, their patterns are compared, and multiple aspects of the disease are studied.

The analysis of SEER data suggests that racial differences exist among the six major subtypes of melanoma in the U.S. in terms of patients’ clinicopathologic characteristics, incidence, treatment, and survival. The observed differences vary across subtypes. Some plausible causes of such differences are provided. SEER data may be limited by lacking certain important information. More comprehensive data collection is needed to fully decipher the racial differences. Despite certain limitations, the findings of this study can be important for early detection, risk stratification, proper treatment selection, and elimination of racial disparities in melanoma.

## Abbreviations

ALM: Acral lentiginous melanoma malignant
Asian/PI: Asian and Pacific Islanders
HW: Hispanic white
LMM: Lentigo maligna melanoma
MIS: Melanoma in situ
NHW: Non-Hispanic white
NM: Nodular melanoma
NOS: Malignant melanoma NOS
SSM: Superficial spreading melanoma
